# Perturbing the Cellular Levels of Steroid Receptor Coactivator-2 Impairs Murine Endometrial Function

**DOI:** 10.1371/journal.pone.0098664

**Published:** 2014-06-06

**Authors:** Maria M. Szwarc, Ramakrishna Kommagani, Jae-Wook Jeong, San-Pin Wu, Sophia Y. Tsai, Ming-Jer Tsai, Bert W. O’Malley, Francesco J. DeMayo, John P. Lydon

**Affiliations:** 1 Department of Molecular and Cellular Biology, Baylor College of Medicine, Houston, Texas, United States of America; 2 Department of Obstetrics, Gynecology and Reproductive Biology, Michigan State University, College of Human Medicine, Grand Rapids, Michigan, United States of America; Baylor College of Medicine, United States of America

## Abstract

As pleiotropic coregulators, members of the p160/steroid receptor coactivator (SRC) family control a broad spectrum of transcriptional responses that underpin a diverse array of physiological and pathophysiological processes. Because of their potent coregulator properties, strict controls on SRC expression levels are required to maintain normal tissue functionality. Accordingly, an unwarranted increase in the cellular levels of SRC members has been causally linked to the initiation and/or progression of a number of clinical disorders. Although knockout mouse models have underscored the critical non-redundant roles for each SRC member *in vivo*, there are surprisingly few mouse models that have been engineered to overexpress SRCs. This deficiency is significant since SRC involvement in many of these disorders is based on unscheduled increases in the levels (rather than the absence) of SRC expression. To address this deficiency, we used recent mouse technology that allows for the targeted expression of human SRC-2 in cells which express the progesterone receptor. Through cre-loxP recombination driven by the endogenous progesterone receptor promoter, a marked elevation in expression levels of human SRC-2 was achieved in endometrial cells that are positive for the progesterone receptor. As a result of this increase in coregulator expression, female mice are severely subfertile due to a dysfunctional uterus, which exhibits a hypersensitivity to estrogen exposure. Our findings strongly support the proposal from clinical observations that increased levels of SRC-2 are causal for a number of endometrial disorders which compromise fertility. Future studies will use this mouse model to decipher the molecular mechanisms that underpin the endometrial defect. We believe such mechanistic insight may provide new molecular descriptors for diagnosis, prognosis, and/or therapy in the clinical management of female infertility.

## Introduction

From mammary morphogenesis to reproduction, energy metabolism to circadian rhythms, members of the p160/Steroid Receptor Coactivator (p160/SRC) family act as pleiotropic integrators of a wide spectrum of signaling cues that drive both physiological and pathophysiological processes [1]. While sharing strong sequence homology, each of the three members of the p160/SRC family (SRC-1, -2, and -3) exert non-redundant roles *in vivo*. Reflecting their complex functional domain structure and pleiotropic functionality, the SRCs can interact with numerous members of the nuclear receptor superfamily of transcription factors as well as with a myriad of other transcription factors involved in cell-fate specification, proliferation, and/or differentiation [2].

Given their diverse coregulator properties, it is not surprising that dysregulation of SRC expression levels has been causally linked to numerous clinicopathologies, ranging from cancers of the breast and prostate to metabolic dysfunction and endometriosis [3]–[9]. Along with clinical data, knockout mouse models for each SRC (or combinations thereof) have been routinely used to support coregulator involvement in both the normal function and etiopathogenesis of a given target tissue [10]–[16]. However, as valuable as these knockout mouse models are in assessing whether absence of a particular SRC alters disease progression, these mice fail to model SRC overexpression, a frequent molecular underpinning for many of these pathologies [3], [4], [17]–[21]. Given the aforementioned, there are surprisingly few mouse models that have been engineered to overexpress a SRC. To date, these models have been designed to target the overexpression of just one SRC member (SRC-3 (NCOA-3; AIB-1) or its variant) to one target tissue: the mammary gland, using the mouse mammary tumor virus (MMTV) promoter [22]–[25].

In the case of SRC-2 (also known as NCOA2, TIF-2, and GRIP-1), both a global and a cell-type specific knockout of SRC-2 in the mouse underscored a pivotal role for this coregulator in endometrial decidualization [5], [26], [27], a normal cellular transformation process required for embryo implantation into the maternal compartment [28]. As with other target tissues, aberrant overexpression of SRC-2 (along with SRC-3) in endometrial tissue has been associated with its dysfunction. For example, clinical studies reveal that SRC-2 and SRC-3 levels are elevated in endometrial biopsies from patients with polycystic ovary syndrome (PCOS) [29]–[31]. Significantly, the endometrium of PCOS patients displays severe defects in functionality, which includes increased miscarriage rates and endometrial cancer susceptibility [32]–[35]. In separate studies, elevated transcript levels of SRC-2 (in addition to SRC-1 and SRC-3) have been detected in the hyperplastic and neoplastic human endometrium [36]–[39]. Though descriptive, these clinical findings propose a causal link between elevated expression of SRC-2 (as well as other SRC members) and the emergence of these endometrial disorders. While this proposal has compelling clinical support, it has yet to be directly tested using a mouse model which is designed to elevate SRC-2 expression levels.

To address this issue, we describe the generation of a new mouse model which is engineered to conditionally express epitope-tagged human SRC-2 at levels significantly higher than those observed for the endogenous murine ortholog. Using this model, we demonstrate that elevation of endometrial SRC-2 levels results in a marked subfertility defect which is based on an inability of the endometrium to fully decidualize. Apart from providing critical *in vivo* support for the involvement of SRC-2 overexpression in endometrial dysfunction, we predict this model will be an important investigative tool to define the key molecular mechanisms that underlie this dysfunctionality in the future.

## Materials and Methods

### Generation of the PR^cre/+^/SRC-2^LSL/+^ (SRC-2:OE) Mouse

Using established methods [40], at least three separate mouse embryonic stem cell lines (R1 [41]) carrying a single copy of a minigene – comprising (in the 5′ to 3′ direction) the CAGGS promoter, a LoxP-STOP-LoxP (LSL) cassette, a cDNA encoding epitope-tagged human (*h*) SRC-2, and a polyadenylation signal – were used to generate the monogenic *h*SRC-2^LSL/+^ mouse [42], [43]. The ubiquitously active CAGGS hybrid promoter consists of the chicken β-actin promoter and the cytomegalovirus (CMV) enhancer [44]. The epitope-tag comprises a tandem FLAG-Myc tag fused in-frame to the 5′ end of *h*SRC-2 whereas the 3′ rabbit globin polyadenylation signal serves as the polyadenylation signal for the minigene [43]. Using a strategy previously described [42], the minigene was targeted to the ROSA26 locus by homologous recombination. Although the CAGGS promoter is active in most tissues [45], placing the LSL cassette between the CAGGS promoter and the downstream *h*SRC-2 transgene prevents expression of the latter. Tissue or cell-type specific expression of the tagged-*h*SRC-2 (driven by the CAGGS promoter) is enabled when the LSL cassette is excised by a cre recombinase driven by a specific promoter of choice, for these studies, the endogenous promoter of the progesterone receptor (PR) [46].

To generate the bigenic PR^cre/+^SRC-2^LSL/+^ mouse, which is designed to target expression of epitope-tagged *h*SRC-2 at high levels in cells that express the PR, the PR^cre/+^ mouse [46] was crossed with a number of *h*SRC-2^LSL/+^ lines. For studies described here, mice carrying alleles for the PR^cre^ knockin and the CAGGS-*h*SRC-2^LSL^ minigene were maintained as heterozygotes in a mixed 129SvEv/C57BL6J background. From hereon, the PR^cre/+^SRC-2^LSL/+^ bigenic is referred to as the SRC-2:OE (overexpressor) mouse, whereas the monogenic *h*SRC-2^LSL/+^ control sibling is abbreviated to SRC-2^LSL^. Embryonic stem cell lines and mice were genotyped by Southern and PCR methods as previously described [40]. The SRC-2 knockout mouse in a C57BL6 background was used as a negative control [11].

### Ethics Statement

All mouse studies were conducted in accordance with the Guide for the Care and Use of Laboratory Animals published by the National Institutes of Health and animal protocols approved by the Institutional Animal Care and Use Committee (IACUC) of Baylor College of Medicine under protocol numbers: AN-1513, AN-544, and AN-4203.

### Animal Husbandry

Mice were housed in a 12 h light/dark photo-cycle at 22°C (±2°C) and received irradiated Teklad global soy protein-free extruded rodent diet (Harlan Laboratories, Inc., Indianapolis, IN) and fresh water *ad libitum*. Surgeries were performed using avertin anesthesia (2.5% (v/v) solution, 0.02 ml/g body weight) and post-operative pain was alleviated with the analgesic, ketoprofen (5–10 mg/kg of body weight). Two hours prior to euthanasia, mice received an intraperitoneal injection (I.P.) of 5-bromo-2′-deoxyuridine (BrdU; Amersham Biosciences, Piscataway, NJ) at a dose of 1 mg per 20 g body weight.

### Staging the Mouse Estrous Cycle

To avoid an artificial estrus response, epithelial cells were gently flushed from the vaginal cavity with 0.1 ml of sterile phosphate buffered saline (PBS) [47]. The cell lavage was evenly spread on a glass slide and dried at room temperature to affix cells. Vaginal smears were rehydrated with water and stained with Harris hematoxylin and eosin phloxine stain (Poly Scientific, Bay Shore, NY) followed by washes with increasing concentrations of ethanol and xylene before mounting with permount and cover slipped (Fisher Scientific, Pittsburgh, PA). Each of the four stages of the estrous cycle (*i.e.* proestrus, estrus, metestrus, and diestrus) were determined as described [47].

### Mammary Gland Whole Mount

Inguinal (#4) mammary glands were fixed overnight in ethanol: acetic acid (3∶1), rehydrated with 70% ethanol and water before staining in carmine aluminum stain for 18 hours. Mammary fat pads were then cleared in toluene prior to mounting on slides with permount (Fisher Scientific, Pittsburgh, PA) [48].

### Steroid Hormone Treatments

For the decidual response assay, mice were ovariectomized at six weeks-of-age and rested for two weeks to purge endogenous ovarian hormones. After the two week rest period, mice daily received estradiol-17β (E2; Sigma, St. Louis, MO) by subcutaneous (s.c.) injection for three days (100 ng/100 µl sesame oil per day). After two days of rest, mice were injected s.c. with progesterone (P4; Sigma, St. Louis, MO) and E2 (1 mg/day and 6.7 ng/day respectively). On the third day of E2P4 treatment, the left uterine horn of each mouse was instilled with 100µl of sesame oil to elicit the deciduogenic response [49]. For these studies, therefore, the left horn is referred to as the stimulated (S) horn whereas the right is designated the control (C) horn. Following oil instillation, mice continued to receive a daily E2P4 injection for an additional five days (day eight of E2P4 treatment). Tissue for histological analysis was taken from the midsection of the uterine horn and fixed overnight at 4°C in 4% paraformadehyde (Electron Microscopy Sciences, Hatfield, PA) in PBS. Following fixation, tissues were washed with 70% ethanol, dehydrated with increasing concentrations of ethanol and xylene before embedding in paraffin [40]. The remaining uterine tissue was snap frozen at −80°C for molecular analysis.

To assess comparative responsiveness of the uterus to short-term E2 exposure, mice were ovariectomized at six weeks-of-age and rested for two weeks before receiving a daily s.c. injection of either E2 (100 ng/100 µl of sesame oil) or 100µl of sesame oil (vehicle control). Seventy two hours after the first injection, mice were euthanized and uterine tissue processed for analysis.

For superovulation studies, four week old mice were I.P. injected with 5 international units (I.U.) of pregnant mare serum gonadotropin (PMSG; VWR Scientific, West Chester, PA), followed 48 hours later by an injection of 5 I.U. of human chorionic gonadotropin (hCG or Pregnyl, Organon International, Roseland, NJ). Following an additional 24 hours, mice were euthanized and oviducts flushed for oocyte isolation and counted using a Nikon SMZ-2B stereo-zoom microscope [40].

### Serum Hormone Measurements

Blood was collected through retro-orbital bleeding and allowed to clot in Microtainer tubes with serum separators (Becton Dickinson, Franklin Lakes, NJ) before centrifugation at 2000×g for 15 minutes to isolate the serum. Serum levels of P4 and E2 were measured by radioimmunoassay and ELISA respectively by the Ligand Assay and Analysis Core at the University of Virginia Center for Research in Reproduction, Charlottesville, VA.

### Immunohistochemical Analysis

Human and mouse tissue sections (5 µm thickness) were deparafinized with xylene and rehydrated with sequential ethanol and water washes. Antigens were unmasked at high temperature with a citrate-based antigen unmasking solution (Vector Laboratories, Burlingame, CA). Endogenous peroxidase activity was blocked by incubating the samples in 3% hydrogen peroxide in methanol. Non-specific staining was blocked with Tyramide Signal Amplification (TSA) system blocking buffer (PerkinElmer Inc., Waltham, MA) before tissue sections were incubated overnight at 4°C with primary antibodies diluted in TSA. The primary antibodies for the following antigens were used: myc-tag (1∶200 dilution, Cell Signaling Technology, Danvers, MA), BrdU (1∶10 dilution, GE Healthcare Biosciences, Pittsburgh, PA), SRC-2 (TIF-2) antibody (1∶300 dilution, BD Biosciences, San Jose, CA), and phospho-histone H3 (1∶500 dilution, Millipore, Billerica, MA). Following the primary antibody incubation, samples were incubated sequentially with biotinylated goat anti-rabbit or anti-mouse IgG (Vector Laboratories Burlingame, CA, 1∶200 dilution) for 1 h and subsequently with VECTASTAIN Elite horseradish peroxidase conjugated avidin (Vector Laboratories Burlingame, CA) for 30 min at room temperature. Antigens were detected by incubation with DAB Peroxidase Substrate Kit (Vector Laboratories Burlingame, CA) for a time period (usually 3–5 min) sufficient to yield a visible brown color. Samples were counterstained with Harris hematoxylin (Poly Scientific, Bay Shore, NY) followed by washes with increasing concentrations of ethanol and xylene. Samples were mounted with permount (Fisher Scientific, Pittsburgh, PA) before being coverslipped. Immuno-detection of murine SRC-2 was performed with mouse SRC-2 (TIF-2) antibody (diluted 1∶300, BD Biosciences, San Jose, CA) using Mouse on Mouse (M.O.M.) kit according to manufacturer’s instructions (Vector Laboratories, Burlingame, CA).

Slides containing a tissue microarray comprising 59 cores (2 mm diameter; 4 µm thickness) of normal human endometrial tissue (biopsied at the proliferative and secretory stage of the menstrual cycle) were obtained from Imgenex (Novus Biologicals) San Diego, CA (HISTO-Array; IMH-139).

To determine the percentage of endometrial epithelial cells that is immunopositive, five SRC-2^LSL^ mice and an equal number of SRC-2:OE mice were used. The average number of immunopositive cells (*i.e.* BrdU or phosphohistone H3 positive) was estimated from a total of 500 epithelial cells counted from four separate transverse uterine sections per mouse (five mice per genotype and treatment). Final counts were expressed as an average percentage (± standard deviation (S.D.)) of total epithelial (luminal or glandular) cells counted. To estimate the average diameter (mm) of the uterine horn, digital images were taken at low magnification (50X) of three intact transverse sections of both uterine horns (denoted horn 1 and 2) per mouse (five mice per genotype were included). The first diameter (A) was measured (using Image J software; http://rsbweb.nih.gov/nih-image/) from two diametrically opposed points, each point located at the external perimeter of the longitudinal smooth muscle layer. A second diameter (B) measurement was taken perpendicular to the first diameter (A) measurement (the average of the two measurements (A and B) was estimated per uterine section). Final diameter measurements were expressed as an average (mm) from five mice per genotype.

Images were captured using a color chilled AxioCam MRc5 digital camera attached to a Carl Zeiss AxioImager A1 upright microscope (Zeiss, Jena, Germany); digital images were processed and assembled using Adobe Photoshop version CS6 (Adobe Systems, San Jose, CA).

### Western Blot

Uterine tissue was homogenized on ice in Tris-Triton-X100 tissue lysis buffer containing complete mini protease inhibitors (Roche, Basel, Switzerland). The tissue lysate was then incubated on ice for 20 min before the protein supernatant was obtained following centrifugation. Protein concentration was determined by the Bradford assay method (Biorad Laboratories, Hercules, CA). Protein (15 µg/lane) was resolved by a 4–15% gradient SDS-PAGE (Biorad Laboratories, Hercules, CA) before transfer to a polyvinylidene fluoride membrane (Millipore, Billerica, MA) [50]. Non-specific IgG binding was blocked with 5% milk in Tris-buffered saline (TBS) with 0.1% Tween. Immunoreactive bands were detected with the following antibodies: FLAG-tag (1∶1000 dilution, Sigma-Aldrich, St. Louis, MO), myc-tag (1∶1000 dilution,OriGene Technologies, Rockville, MD), SRC-2 (1∶2000 dilution, Bethyl Laboratories, Inc., Montgomery, TX), and β-actin (1∶10000 dilution, loading control; Sigma-Aldrich, St. Louis, MO). The immunoreactive bands were detected with horseradish peroxidase-conjugated goat anti-rabbit IgG or goat anti-mouse IgG (Santa Cruz Biotechnology, Santa Cruz, CA) and visualized with SuperSignal West Pico Chemiluminescent Substrate (Thermo Scientific, Rockford, IL).

### Real-time PCR Gene Expression Analysis

Total RNA was isolated from uterine tissues with TRIzol reagent according to manufacturer’s instructions (Life Technologies, Carlsbad, CA). Isolated total RNA was incubated with DNase I and further purified using RNeasy spin columns (Qiagen, Valencia, CA). From 1 ug of total RNA, cDNA was prepared using the SuperScript VILO cDNA Synthesis Kit (Life Technologies, Carlsbad, CA) before real-time PCR analysis was performed using TaqMan Universal Master Mix II with validated TaqMan Gene Expression Assays (Life Technologies, Carlsbad, CA). Resultant amplicons were quantitated using a QuantStudio 12 K Flex Real-Time PCR System; 18S rRNA was used as an endogenous control. The TaqMan Gene Expression Assays are listed in **Table S1**.

### Statistical Analysis

Unless otherwise stated, statistical analyses were performed with one-way ANOVA followed by two-tailed independent sample Student’s *t*-tests or Welch two-sample *t*-test in RStudio with the R Commander package (RStudio, Inc., Boston, MA). Non specific (n.s.): p>0.05; *: p≤0.05; **: p≤0.01; and ***: p≤0.001.

## Results

### The Spatial Profile and Level of Endometrial SRC-2 Expression are not Markedly Altered during the Murine Estrous Cycle

**Figure 1:**
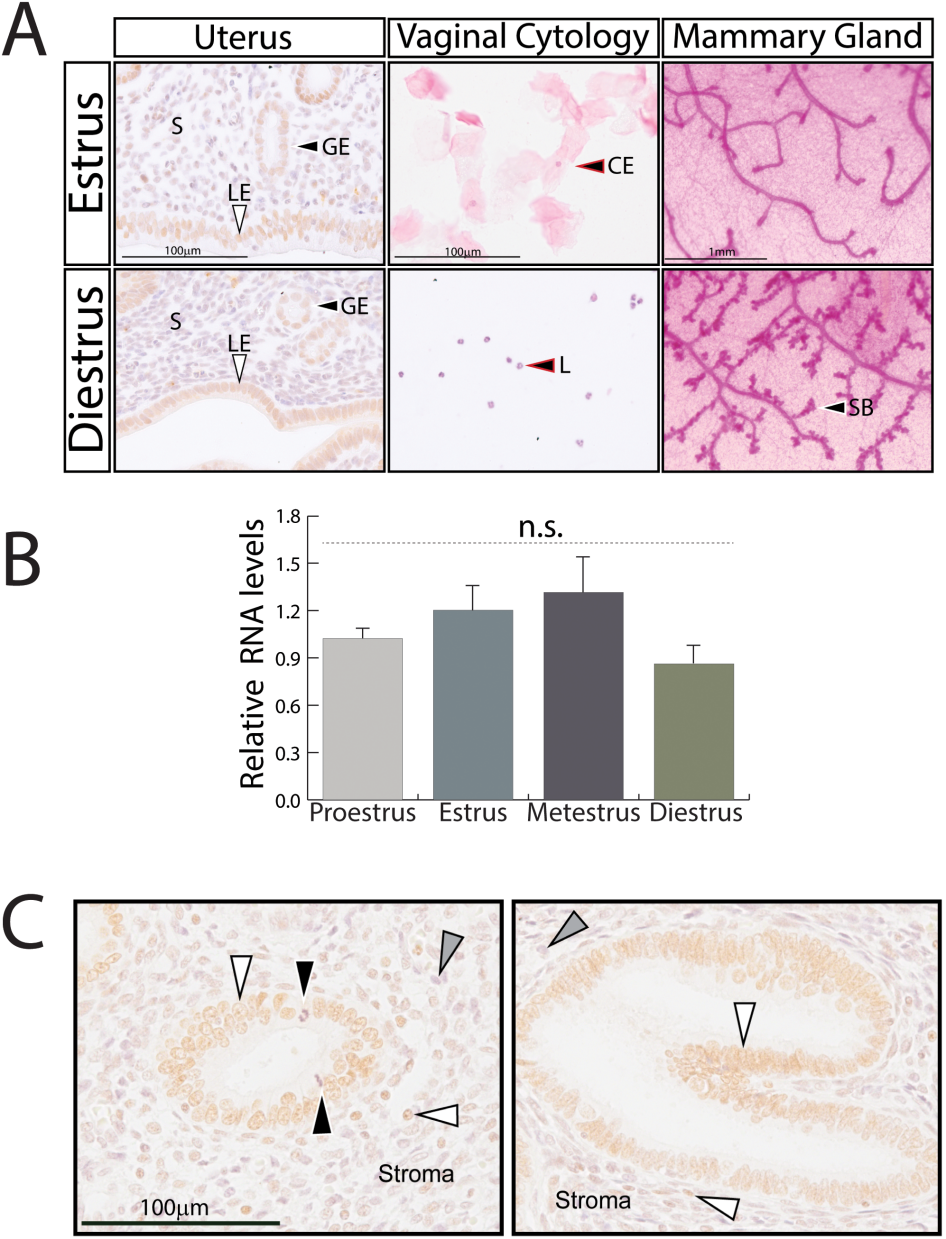
Expression levels of SRC-2 in the murine endometrium are not significantly altered during the estrous cycle. (A) Left panels show immunostaining of SRC-2 in the murine endometrium during the estrus (top panel) and diestrus (bottom panel) phase of the estrous cycle; S, GE, and LE denote stroma, glandular epithelium, and luminal epithelium respectively. Note the slightly edematous stromal compartment and columnar luminal epithelial cells typical of an endometrium during the estrus stage of the murine cycle [67]. Conversely, luminal epithelial cells are more cuboidal and the sub-epthelial stroma is more condensed in the endometrium at diestrus. Middle panels show the corresponding vaginal cytology (cornified squamous epithelial (CE) cells and polymorphonuclear leukocytes (L)) which confirm the stage of the cycle [47], [52]. Right panels show corresponding mammary gland whole mounts; SB indicates side-branches. Note: a transient increase in the number of ductal side branches in the mammary gland is known to occur during the diestrus phase of the mouse estrous cycle [51]. Scale bar in top panels apply to corresponding bottom panels. (B) Comparative SRC-2 transcript levels in the murine endometrium at each of the four stages of the estrous cycle; n.s. denotes non-specific. (C) Immunostaining of SRC-2 in the human endometrium biopsied during the proliferative (left panel) and secretory (right panel) stages of the menstrual cycle. White arrowhead indicates glandular epithelial cells and stromal cells positive for SRC-2 expression; black arrowhead in left panel shows mitotic figures whereas the grey arrowhead in both panels indicates stromal cells negative for SRC-2 expression. Note the tortuosity typically exhibited by the glandular epithelium during the secretory phase of the menstrual cycle [68] (**Fig. 1C** (right panel)). Scale bar in the left panel applies to the right panel.

Immunohistochemistry and quantitative real-time PCR analysis were performed to determine whether endometrial SRC-2 expression levels change during the murine estrous cycle and whether its expression shifts from one endometrial cell-type to another with cycle progression. As observed in **Fig. 1A**, the spatial expression profile and levels of SRC-2 expression are indistinguishable in the endometrium of a wild type mouse at the estrus and diestrus stage of the estrous cycle; vaginal cytology and mammary gland morphology confirmed each stage of the cycle [47], [51], [52] (proestrus and metestrus stages showed similar results (data not shown)). In the case of the estrus phase of the cycle, SRC-2 expression is pronounced in the luminal and glandular epithelium as compared to the edematous stromal compartment. Whereas the majority of epithelial cells score positive for SRC-2 expression, not every stromal cell is immunopositive for SRC-2 (**Fig. 1A** (left top panel)). A similar spatial profile and level of expression for endometrial SRC-2 is observed at the diestrus stage of the cycle (**Fig. 1A** (left bottom panel)). These results indicate that endometrial SRC-2 protein levels are not significantly modulated by cycling hormone (*i.e.* E2 and P4) levels which occur during the cycle [53]. However, it is important to note that these results do not address whether post-translational modifications (*i.e.* phosphorylation) of the coregulator change with cycle progression; post-translational modifications are known regulators of SRC activity in other physiological systems [54]. In **Fig. 1B**, quantitative real-time PCR analysis demonstrates that endometrial SRC-2 transcript levels do not change with each stage of the cycle in the wild type mouse, further supporting conclusions drawn from results shown in **Fig. 1A**. Importantly, similar findings are obtained by immunohistochemical analysis of human endometrial samples biopsied during the proliferative and mid-secretory phase of the menstrual cycle (**Fig. 1C**). Like the mouse, SRC-2 expression is predominantly expressed in the epithelium of the human endometrium with equivalent expression levels of SRC-2 during the proliferative and secretory phases of the cycle; these findings confirm previous clinical observations [29]. Together, these results support the conclusion that transcript and protein levels of endometrial SRC-2 are not markedly altered during murine cycle progression, with a similar SRC-2 expression profile observed in the human endometrium.

### Generation of a Mouse Model that Expresses Human SRC-2 at Elevated Levels

**Figure 2:**
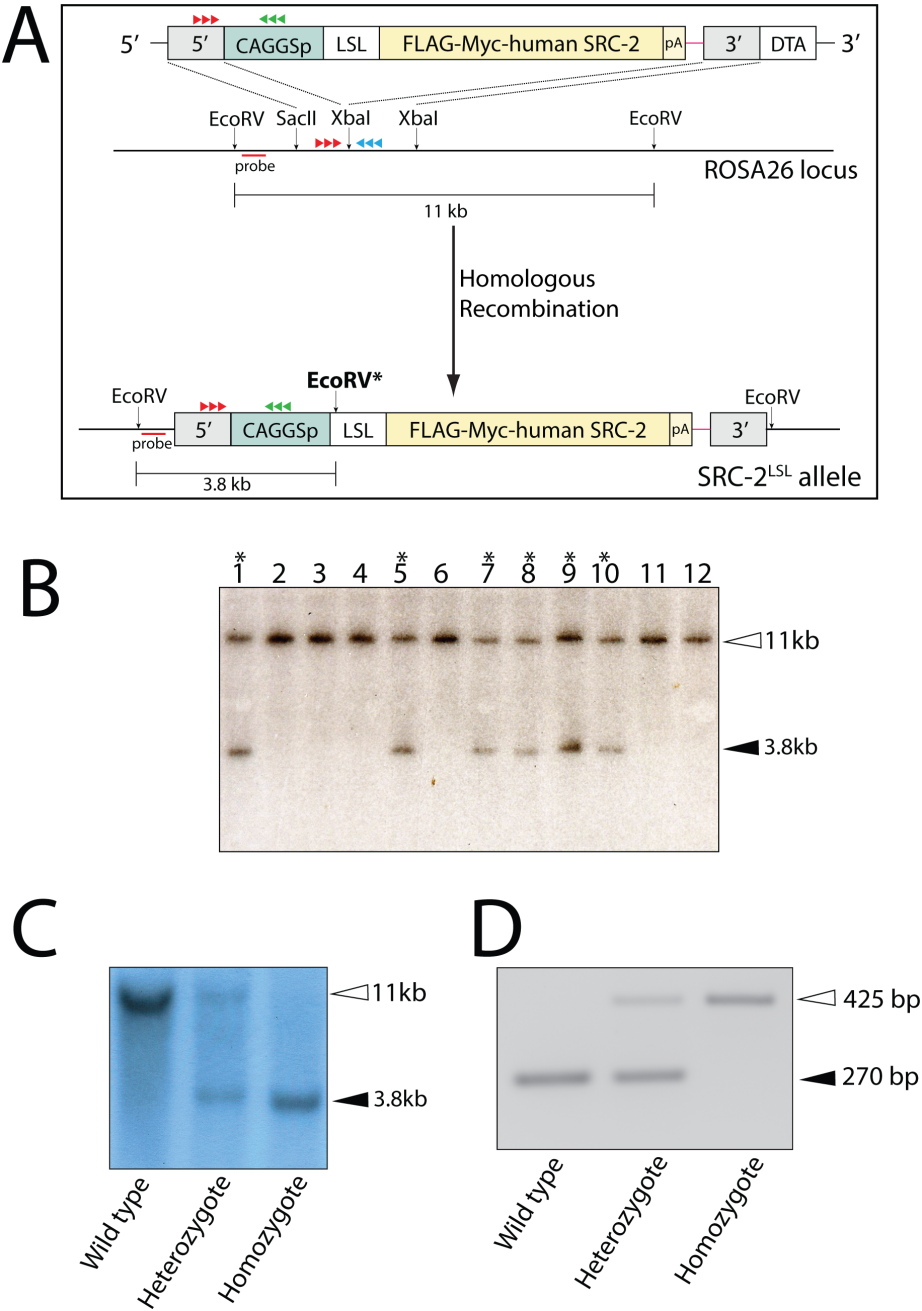
Generation of the SRC-2^LSL^ mouse. (A) Schematic of the targeting strategy to introduce the SRC-2^LSL^ transgene into the ROSA26 locus by homologous recombination in murine ES cells. The targeting vector containing the epitope-tagged SRC-2^LSL^ transgene was targeted to a region of the ROSA26 locus which is flanked by endogenous EcoRV sites that are 11 kb apart. Introduction of the targeting vector into the ROSA26 locus introduces an additional internal EcoRV (bold); probe (red line) indicates the location of the 5′ probe used for Southern analysis whereas red and green arrowheads indicate the location of the PCR primers used for genotyping. (B) Representative Southern blot result of ES cell genomic DNA digested with EcoRV and probed with the 5′ probe (panel (A)). Six targeted events (lanes 1, 5, and lanes 7–10) as indicated by the 3.8 kb hybridizing band are shown. (C) Southern result of genomic DNA from tail biopsies from F1 generation mice digested with EcoRV and probed with the 5′ probe (panel (A)). Wild type mice exhibit the single 11 kb hybridizing band (non-targeted endogenous ROSA26 locus (panel (A)); mice heterozygous for the targeted event display both the 11 kb and 3.8 kb hybridizing band whereas a single 3.8 kb hybridizing band indicates mice homozygous for the targeted event. (D) The amplicon sizes from PCR genotyping of wild type mice as well as mice heterozyogous and homozygous for the targeted event is shown (PCR primers (panel A) generate the 270 bp amplicon from the endogenous ROSA26 locus or a 425 bp amplicon from the targeted allele).

**Figure 3:**
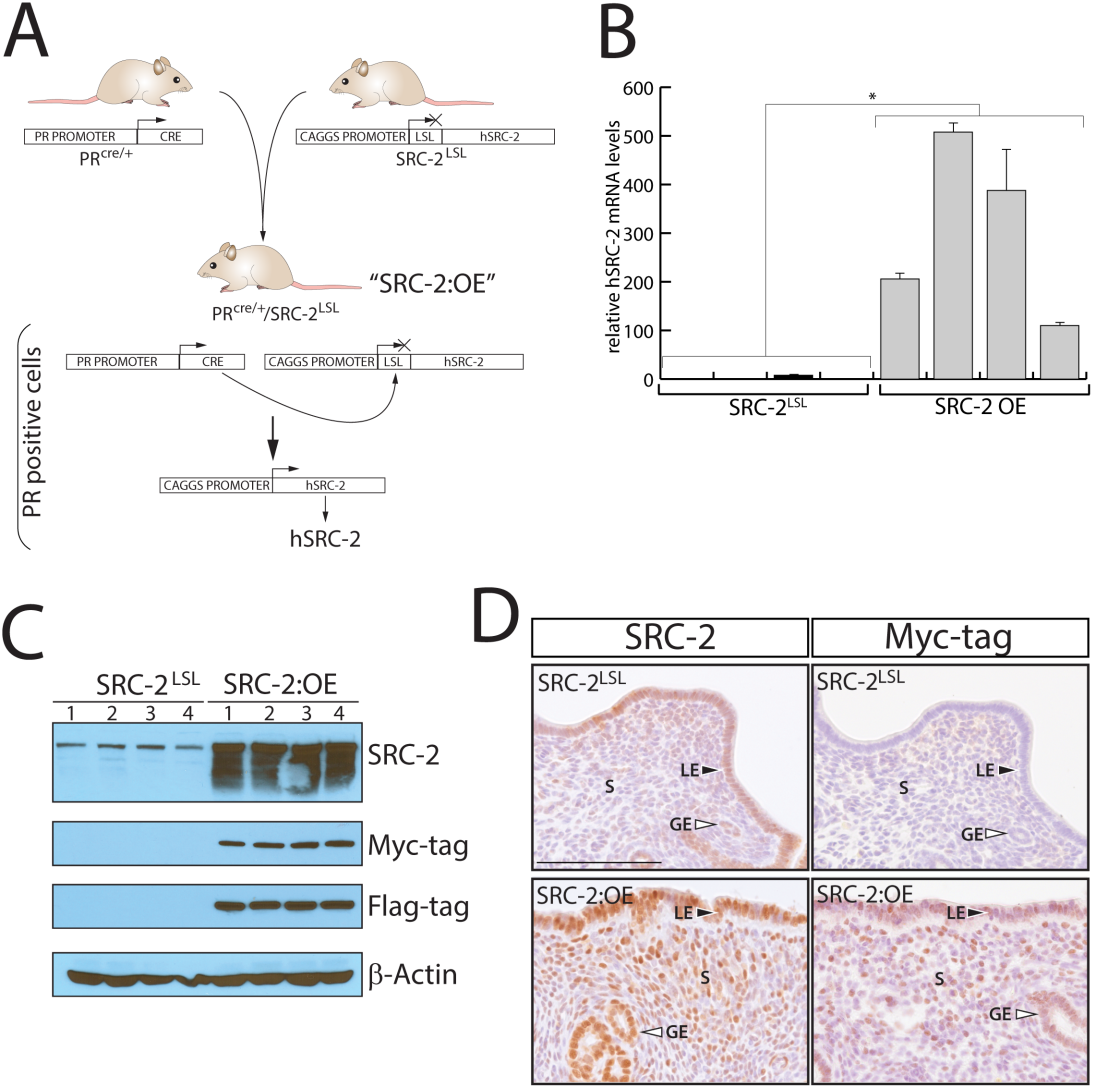
The SRC-2:OE bigenic mouse expresses high levels of human SRC-2 in the endometrium. (A) The SRC-2:OE mouse was generated by crossing our PR^cre/+^ mouse [46] with the SRC-2^LSL^ mouse (**Fig. 2**); the SRC-2:OE mouse is heterozygous for the PR^cre^ and SRC-2^LSL^ alleles. The cre/loxP genetic design selectively targets *h*SRC-2 expression at high levels in PR positive cells of the SRC-2:OE mouse. (B) Transcript levels of endometrial *h*SRC-2 in the SRC-2:OE mouse as compared to the SRC-2^LSL^ monogenic control are shown (*p<0.05). (C) Western analysis shows high levels of SRC-2 protein expression in the SRC-2:OE endometrium as compared to the SRC-2^LSL^ control; each lane represents pooled samples from five mice. The immunoblot also shows specific expression of the myc and Flag epitope tags in the SRC-2:OE lanes; β-actin is a loading control. (D) Immunohistochemical staining for SRC-2 and the myc-tag reveals that the *h*SRC-2 transgene is expressed in the luminal epithelium (LE) and glandular epithelium (GE) as well as in the stromal (S) compartment of the SRC-2:OE endometrium. Scale bar applies to all panels.

As a first step toward generating a mouse model in which human SRC-2 is expressed at elevated levels in the uterus, a mouse harboring the *h*SRC-2^LSL^ minigene at the ROSA26 locus was generated from targeted mouse embryonic stem cells using a homologous recombination strategy detailed in **Fig. 2A**. With a targeting efficiency of 42% (40/96 total ES cells screened; (**Fig. 2B**)), at least three targeted ES cell clones were used to successfully generate the SRC-2^LSL^ mouse through the germline (**Fig. 2C and D**). To generate the SRC-2:OE mouse, our PR^cre/+^ mouse [46] was crossed with the *h*SRC-2^LSL^ mouse line to create the PR^cre/+^/*h*SRC-2^LSL^ or SRC-2:OE bigenic (**Fig. 3A**). Through cre-mediated excision, the SRC-2:OE bigenic is engineered to express high levels of *h*SRC-2 (driven by the CAGGS promoter) selectively in cells that express the PR. Quantitative real-time PCR clearly shows that significantly elevated levels of *h*SRC-2 transcripts are generated in the endometrium of the SRC-2:OE mouse as compared to the endometrium of the SRC-2^LSL^ monogenic sibling (**Fig. 3B**). Importantly, transcript levels of endogenous SRC-1, -2, and -3 are not altered as a consequence of introducing elevated levels of *h*SRC-2 in the SRC-2:OE endometrium (**Fig. S1**). Similarly, elevated levels of *h*SRC-2 protein are expressed in the endometrium of the SRC-2:OE mouse as evidenced by western blot analysis using antibodies against *h*SRC-2 (which also cross reacts with endogenous mouse SRC-2) and to the epitope tags encoding Myc and Flag (**Fig. 3C**). Using antibodies to SRC-2 and the Myc epitope tag, immunohistochemistry clearly demonstrates an elevated level of expression for transgene-derived *h*SRC-2 in the luminal and glandular epithelium as well as in the stromal compartment of the SRC-2:OE endometrium (**Fig. 3D**). Importantly, despite expressing elevated levels of *h*SRC-2, the overall health and body weight of the SRC-2:OE mouse is similar to its SRC-2^LSL^ sibling (data not shown).

### Increased Expression Levels of Human SRC-2 in the SRC-2:OE Mouse Enhances Endometrial Epithelial Proliferation

**Figure 4:**
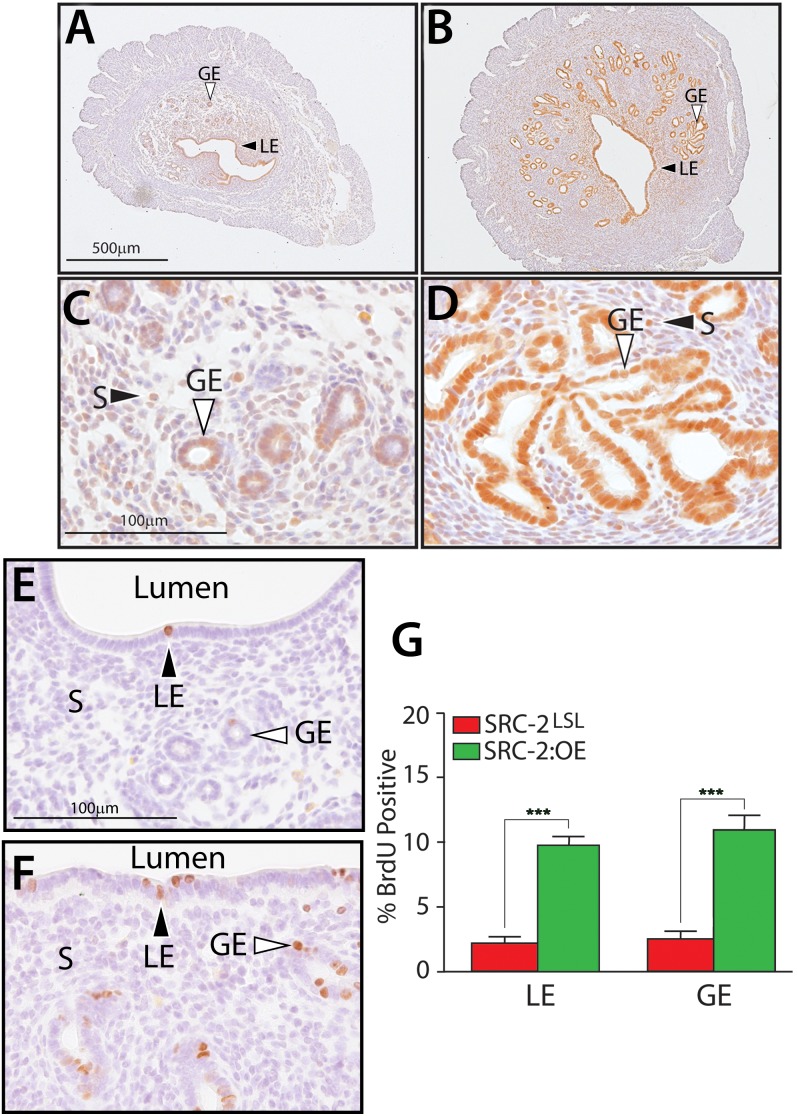
Spatial expression pattern of hSRC-2 in the endometrium of the SRC-2:OE mouse. (A) and (B) represent low magnification images of transverse sections of the uterine mid-horn immunostained for SRC-2 from the SRC-2^LSL^ and SRC-2:OE mouse respectively. (C and D) display higher magnification images of uterine sections shown in (A and B) respectively. Corresponding uterine sections stained for the Myc-epitope tag are shown in **Fig. S2**. (E) and (F) show high magnification images of uterine mid-horn sections stained for BrdU incorporation from the SRC-2^LSL^ and SRC-2:OE mouse respectively. (G) Bar graph shows the percentage of BrdU incorporation in the LE and GE cellular compartments of the SRC-2:OE endometrium compared to the corresponding SRC-2^LSL^ control (***denotes p<0.001 (n = 5 mice per genotype)). Scale bars in (A, C, and E) apply to (B, D, and F) respectively; LE, GE, and S denote luminal epithelium, glandular epithelium, and stroma respectively.

To confirm that the *h*SRC-2 transgene is expressed in all endometrial cell types that express PR and SRC-2 [27], immunohistochemistry was applied to endometrial sections obtained from 3-month old SRC-2:OE and SRC-2^LSL^ siblings (**Fig. 4**). Using antibodies against *h*SRC-2, high levels of *h*SRC-2 expression was detected in the luminal epithelium, glandular epithelium, and stromal cellular compartments of the SRC-2:OE endometrium. As seen at low power magnification, the endometrium of the SRC-2:OE mouse is consistently larger than that of its SRC-2^LSL^ sibling (compare **Fig. 4A** with **4B;** also **Fig. S2**). Although the size of the myometrium is equivalent in the SRC-2:OE and SRC-2^LSL^ uterus, the SRC-2:OE stromal and glandular epithelial compartments are consistently larger than seen in the SRC-2^LSL^ uterus. At higher power magnification, it is clear that the SRC-2:OE endometrium displays larger epithelial glands than the SRC-2^LSL^ endometrium (compare **Fig. 4C** with **4D**; also **Fig. S2.**). The majority of the glandular epithelial cells in the SRC-2:OE endometrium are positive for high levels of *h*SRC-2 expression (**Fig. 4D**; specificity of SRC-2 antibody was confirmed using uterine tissue obtained from the SRC-2 knockout mouse (**Fig. S3)**). Furthermore, immunohistochemical analysis of BrdU incorporation revealed a significant increase in the number of luminal and glandular epithelial cells that score positive for BrdU in the SRC-2:OE endometrium when compared to the SRC-2^LSL^ endometrium (**Fig. 4** **G–I**; also **Fig. S2**). A similar result was observed in six month old SRC-2:OE mice (**Fig. S2**). Given the observed increase in the number of proliferating epithelial cells in the SRC-2:OE endometrium, we next asked whether this increase in proliferation would be enough to influence the fertility status of the mouse.

### The SRC-2:OE Female Exhibits a Severe Subfertility Phenotype

**Figure 5:**
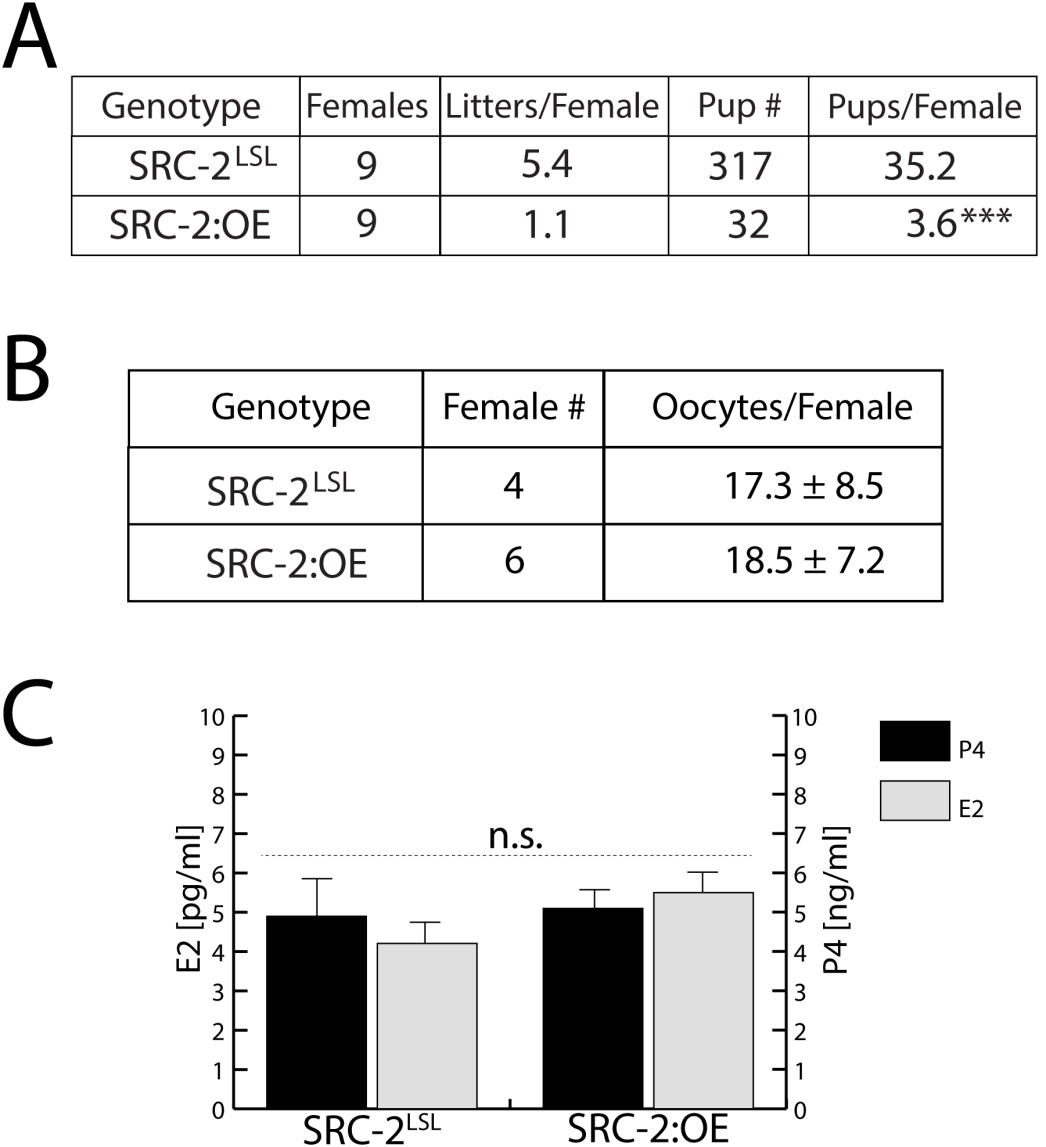
The SRC-2:OE mouse exhibits a severe subfertility defect. (A) A six-month breeding study reveals that the SRC-2:OE female mouse is severely subfertile. (B) SRC-2^LSL^ and SRC-2:OE mice produce similar numbers of oocytes following sequential PMSG and hCG hormone treatments (see: Materials and Methods). (C) Similar levels of E2 and P4 are found in the serum of the SRC-2^LSL^ and SRC-2:OE mouse.

A six month breeding program was employed to determine whether the SRC-2:OE shows a similar fecundity to its SRC-2^LSL^ sibling. The SRC-2:OE mouse exhibits a significant decrease in fertility as evidenced by the small number of litters produced by the SRC-2:OE group when compared to the SRC-2^LSL^ group (**Fig. 5A**). Specifically, four out of nine SRC-2:OE mice failed to produce litters. From the remaining five SRC-2:OE mice, three mice produced one litter whereas the other two females yielded three litters; however, these litters on average were smaller when compared to the control group (average 6.5 pups/litter in the SRC-2^LSL^ group vs. 3.2 pups/litter in the SRC-2:OE group). To assess whether the basis of the subfertility defect is linked (in whole or in part) with ovarian dysfunction, an established gonadotropin hormone regimen was used to elicit superovulation in juvenile SRC-2:OE and SRC-2^LSL^ mice (**Fig. 5B**; Material and Methods [40]). The results in **Fig. 5B** clearly shows that ovarian function in the SRC-2:OE is not compromised; this conclusion is further supported by equivalent serum levels of E2 and P4 in the SRC-2:OE and SRC-2^LSL^ mouse groups (**Fig. 5C**). Collectively, these data reveal a significant subfertility defect is linked to high levels of human SRC-2 expression in the SRC-2:OE; however, the basis of this reproductive phenotype is not due to an anovulatory phenotype.

### The SRC-2:OE Endometrium Exhibits a Limited Ability to Decidualize

**Figure 6:**
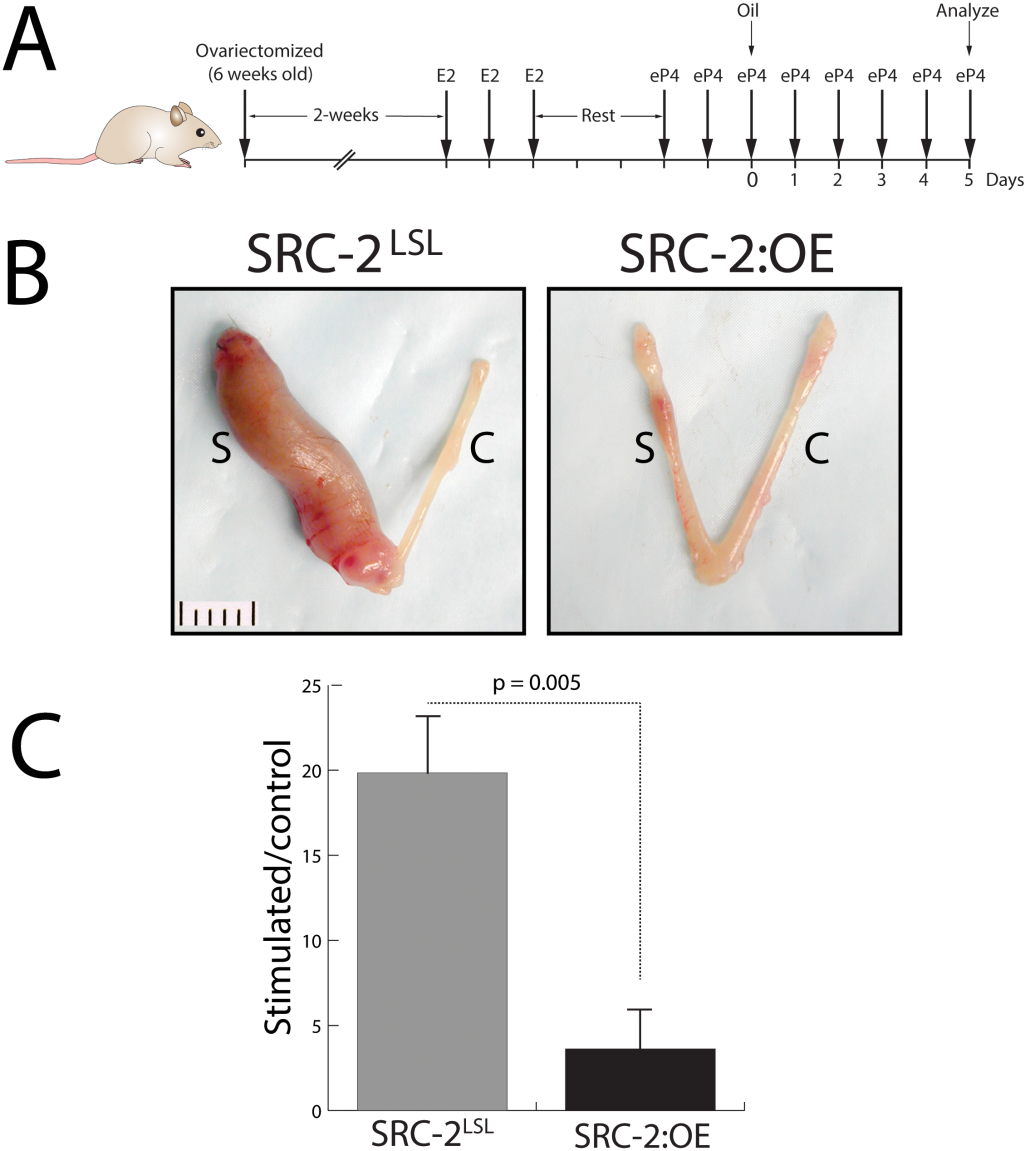
Decidualization is severely impaired in the SRC-2:OE uterus. (A) Schematic showing the hormone regimen used to elicit the decidual response in the uterus of an ovariectomized mouse. (B) Gross morphology of uteri from the SRC-2^LSL^ and SRC-2:OE mouse following the hormone treatment protocol shown in (A); S and C denote stimulated and control horn respectively. Scale bar applies to both panels. (C) Bar graph displays the wet weight ratio of stimulated horn over contralateral control horn for the SRC-2^LSL^ and SRC-2:OE uterus.

To determine whether the subfertility phenotype exhibited by the SRC-2:OE female is caused by a defect in endometrial decidualization, an established hormone treatment protocol applied to ovariectomized mice was used to elicit an endometrial deciduogenic response (**Fig. 6A**). At the gross morphological level, **Fig. 6B and C** clearly show that the signature deciduogenic response is triggered in the stimulated left uterine horn of the SRC-2^LSL^ mouse; however, an equivalent response is not achieved in the similarly treated uterine horn of the SRC-2:OE mouse. These results indicate that perturbing the levels of SRC-2 expression adversely affects the ability of the endometrium to decidualize.

### The SRC-2:OE Endometrium Fails to Display the Critical Cellular and Molecular Changes which Drive Decidualization

**Figure 7:**
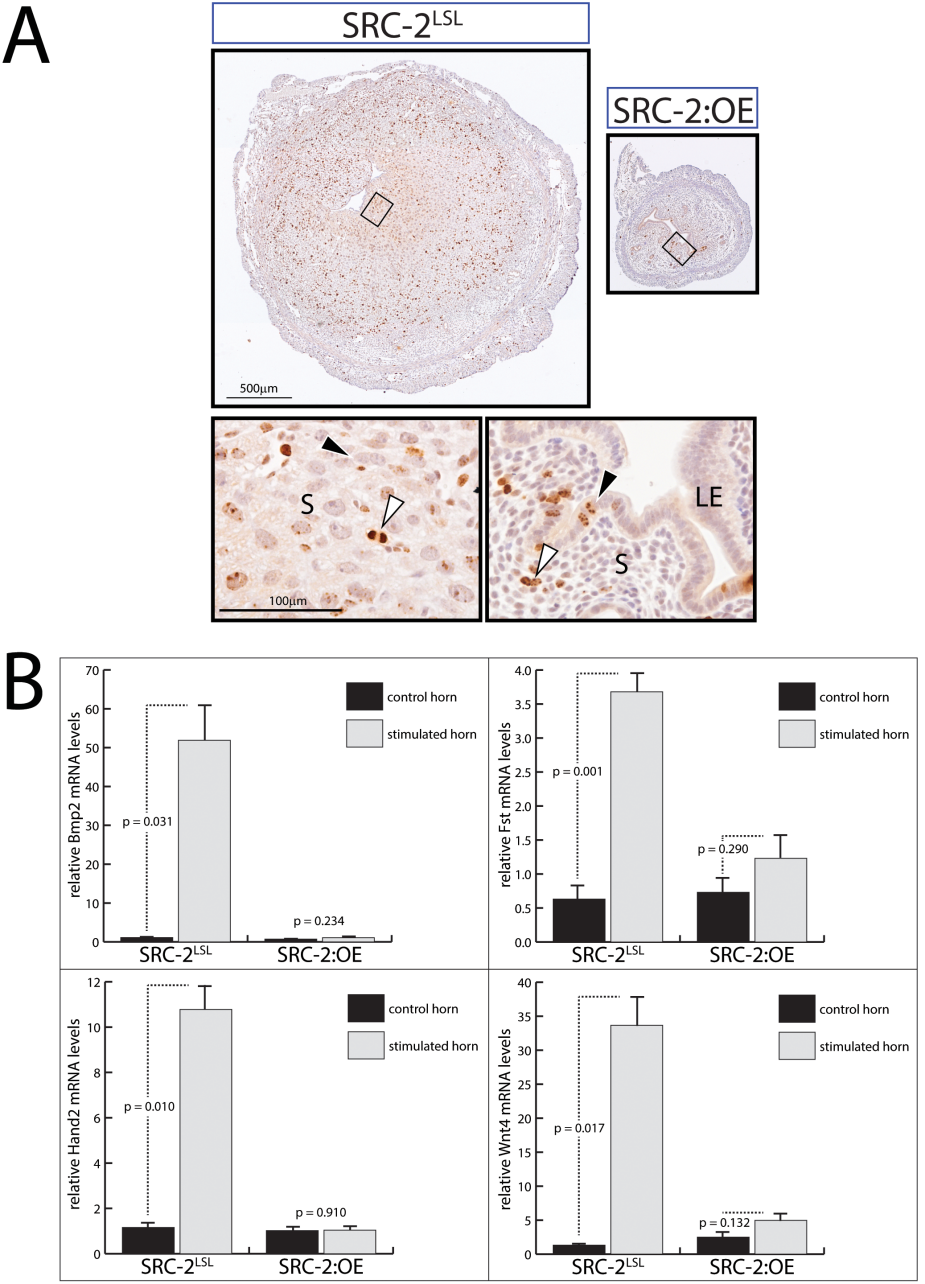
Cellular and molecular analysis of the endometrial decidualization defect in the SRC-2:OE mouse. (A) Top panels show low magnification images of transverse sections of the stimulated (S) uterine horn immunostained for phospho-histone H3 from the SRC-2^LSL^ and SRC-2:OE mouse. Bottom panels are higher magnification images of tissue areas denoted by a box shown in the corresponding top panels. Black arrowhead in bottom left panel indicates a decidual cell; white arrowhead points to a mitotic figure. Black arrowhead in bottom right panel indicates luminal epithelial cells positive for phospho-histone H3 whereas the white arrowhead shows a stromal cell immuno-positive for phospho-histone H3. Note the intact luminal epithelium (LE); S denotes stroma. Scale bar in top and bottom left panels apply to corresponding right panels. (B) Real time PCR analysis of *Bmp2* (Bone morphogenetic protein 2), *Fst* (Follistatin), *Hand2* (Heart and neural crest derivatives expressed transcript 2), and *Wnt4* (Wingless-related MMTV integration site 4) transcript levels in the control and stimulated uterine horns of SRC-2^LSL^ and SRC-2:OE mice.

Comparative histological analysis of the mid-section of the SRC-2^LSL^ and SRC-2:OE stimulated uterine horns clearly underscores the SRC-2:OE decidual defect at the cellular level (**Fig. 7A**). Apart from the striking difference in the size of the respective uterine horns, the SRC-2^LSL^ stromal compartment contains numerous large decidual cells interspersed with a small number of proliferating stromal cells (at this stage of decidualization, the luminal epithelium is absent [55]). In contrast, decidual cells are not detected in the SRC-2:OE stromal compartment (**Fig. 7A**). Strikingly, the luminal epithelial compartment remains intact in the SRC-2:OE endometrium, with a subset of epithelial cells still undergoing active proliferation (**Fig. 7A**). Given the severity of the decidual phenotype, its not surprising that many changes of gene expression previously associated with endometrial decidualization and critical for normal endometrial function are not induced in the stimulated uterine horn of the SRC-2:OE mouse (**Fig. 7B** **, Fig. S5**). Therefore, our results show that a breakdown in the endometrial decidual progression program underpins the subfertility defect displayed by the SRC-2:OE mouse. Future investigations will determine whether embryo attachment to the apical surface of the luminal epithelium (which triggers decidualization in the subepithelial stroma) or a progression step later in the decidual program accounts for the decidualization impairment observed in SRC-2:OE uterus.

### The SRC-2:OE Endometrial Epithelium is Highly Responsive to Estrogen

**Figure 8:**
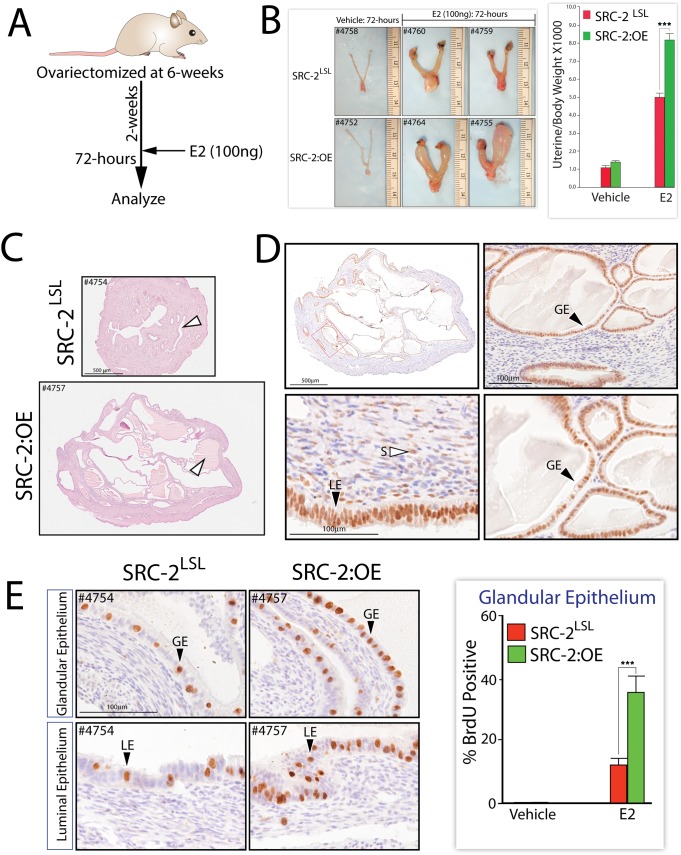
The uterine epithelium of the SRC-2:OE mouse is significantly sensitive to estrogen exposure. (A) Schematic showing the E2 treatment protocol applied to SRC-2^LSL^ and SRC-2:OE mouse groups. (B) Gross morphology of uteri from SRC-2^LSL^ and SRC-2:OE mice following hormone treatment regimen shown in (A). Histogram on the right displays the ratio of uterine weight over body weight for SRC-2^LSL^ and SRC-2:OE mice following vehicle or E2 treatment (***denotes p<0.001 (n = 5 mice per genotype/treatment)). (C) H&E stained uterine mid-horn sections from the SRC-2^LSL^ and SRC-2:OE mice following E2 treatment; white arrowhead shows enlarged endometrial glands; scale bar in top panel applies to bottom panel. (D) Top left panel shows low power magnification image of the uterine mid-section of the SRC-2:OE mouse following E2 treatment stained for the myc-tag (a serial section of H&E stained section shown in bottom panel in (C)). Right top panel is a high power magnification image demarcated by a red box in the top left panel. Two bottom panels are higher power magnification images shown in two top panels; LE, GE, and S denote luminal epithelium, glandular epithelium, and stroma respectively. Scale bar in bottom left panel applies to bottom right panel. (E) High power magnification images of uterine sections stained for BrdU incorporation from SRC-2^LSL^ and SRC-2:OE mice following E2 treatment. Scale bar in top left panel applies to all panels. Histogram in left panel displays the percentage positive for BrdU incorporation in the glandular epithelium of uteri from the SRC-2^LSL^ and SRC-2:OE mouse groups following vehicle or E2 treatment (***denotes p<0.001 (n = 5 mice per genotype/treatment)).

Because the luminal epithelium in the stimulated uterine horn of the SRC-2:OE mouse continues to proliferate following a E2P4 hormone treatment protocol used to elicit decidualization (**Fig. 7**), we asked whether the SRC-2:OE endometrial epithelium is more sensitive to acute E2 exposure than the endometrial epithelium of the SRC-2^LSL^ sibling. An answer to this question also may explain why the endometrial epithelium of the intact SRC-2:OE mouse registers a moderately higher proliferative response than that of the SRC-2^LSL^ sibling (**Fig. 4** and **Figures S2** and **4**). Using a modification of an E2 treatment protocol applied to the ovariectomized mouse [56] (**Fig. 8A**), the SRC-2^LSL^ and SRC-2:OE endometria were assessed at both the gross and cellular level in terms of responsiveness to E2 exposure. At the gross morphological level, **Fig. 8B** clearly shows that the SRC-2:OE uterus exhibits a significantly greater uterotrophic response (*i.e.* an increase in uterine weight due to water imbibition and epithelial proliferation [56]) to E2 administration as compared to the SRC-2^LSL^ control. Histological analysis (H&E as well as Myc-tag staining) of transverse sections of representative mid-uterine horns from the SRC-2:OE and the SRC-2^LSL^ mouse following E2 treatment, clearly shows that the SRC-2:OE endometrium exhibits a more striking uterotrophic response, especially in terms of the size and number of hyperplastic epithelial glands, many of which develop into hemorrhagic cysts (**Fig. 8C and D**). Not surprisingly, immunohistochemical analysis for BrdU incorporation reveals that the endometrial luminal and glandular epithelium of the SRC-2:OE mouse exhibits a significant increase in the number of cells scoring positive for proliferation as compared to the similarly treated SRC-2^LSL^ control mouse (**Fig. 8E**). These results support the proposal that increasing the levels of SRC-2 enhances E2-driven epithelial proliferation in the mouse endometrium, which may explain the subfertility defect exhibited by the SRC-2:OE mouse at the cellular level. Future studies will entail using the SRC-2:OE mouse model to define the molecular mechanisms that are responsible for the endometrial dysfunctional response due to SRC-2 overexpression.

## Discussion

Following nearly two decades of clinical investigation and basic research, it is established that strict controls on SRC expression levels are necessary for normal functioning of various physiologic systems [1]. Accordingly, perturbation (or more specifically, an unscheduled increase) in the cellular levels of one or more members of this coregulator family is known to be a causal factor in the genesis and/or progression of a remarkable array of clinicopathophysiologic processes [3], [4], [9], [22]. Notwithstanding the significant contributions that conventional mouse genetics have made toward our current understanding of the role of SRC family members in normal tissue function and disease progression [8], [27], [57], [58], the majority of these engineered mice fail to model the SRC overexpression phenotype that frequently drives many pathophysiologic states. This insufficiency is further exacerbated by the inability to develop cell lines that stably overexpress SRC family members, thereby denying investigators even a simple *in vitro* model with which to study the effects of SRC overexpression at the cellular level.

Therefore, we have focused our efforts to develop a mouse engineering strategy which enables *in vivo* overexpression of SRC members in specific tissues and cell-types. As proof-of-principle, we describe here the generation of the SRC-2:OE bigenic mouse in which human SRC-2 is expressed at significantly elevated levels in murine PR positive cells using a cre-loxP recombination system. With spatiotemporal specificity defined by the endogenous PR promoter [46], we have successfully targeted overexpression of human SRC-2 to specific cell-types of the uterus that normally express SRC-2, PR, and ER. Generation of such a mouse has allowed us to address a long-standing question in uterine biology: Does elevated expression of one or more SRCs contribute to a dysfunctional uterus? [29].

Our first line of investigations reveals that elevated levels of human SRC in the SRC-2:OE female mouse results in a severe subfertilty defect which is caused by a dysfunctional endometrium. These results along with our recent published studies [5], [27] strongly support the supposition that tight controls of cellular SRC-2 expression levels are critical for normal endometrial functionality. Together, our studies show that both absence of SRC-2 and an unscheduled “ramp-up” of SRC-2 levels leads to a functionally defective endometrium and underscore the importance of maintaining constant levels of SRC-2 protein expression during the mouse and human cycles. Whether similar molecular mechanisms underlie both SRC-2 dependent uterine defects awaits future investigations. Irrespective of the underlying molecular mechanisms, our findings are the first to provide direct experimental support to the earlier clinical proposal that overexpression of SRC family members is causal for an array of endometrial disorders [36]–[39], which include those that compromise fertility [29]–[31].

Hormone treatment of ovariectomized mice indicates that the SRC-2:OE endometrium fails to decidualize and that hypersensitivity to estrogen exposure may be one explanation for this failure. These findings are important as ovarian hyperstimulation (specifically, over sensitization to estrogen) in mice is known to elicit implantation failure and early pregnancy loss [59], [60]. Moreover, elevated estrogen levels arising from ovarian hyperstimulation by prescribed gonadotropin hormones are implicated in producing uterine refractoriness which in turn contributes to a reduction in pregnancy success rates with human *in vitro* fertilization (IVF)/embryo-transfer (ET) protocols that use non-frozen embryos [61]. Interestingly, SRC-2 joins an increasing number of other coregulators (*i.e.* REA, prohibitin, and Ncoa6) which recently have been implicated in modulating the responsiveness of the endometrial epithelium to acute estrogen exposure [62]–[64]. Importantly, we can’t discount a role for SRC-2 overexpression in driving persistent prosurvival signaling in the luminal epithelium of the endometrium which may override local apoptotic signals that are known to be required during embryo implantation [55]. To gain further mechanistic insight, our future studies will use the SRC-2:OE mouse to disclose the direct signaling pathways in the endometrium that are significantly altered by changes in SRC-2 levels particularly during the peri-implantation period.

The persistent epithelial proliferation observed in the SRC-2:OE endometrium leaves open the question of whether this tissue is predisposed to tumorigenesis. Addressing this question is important since overexpression of SRC-2 (as shown for other members of the SRC family [3], [18]–[20], [22], [23], [58], [65]) is linked to the causation and/or progression of a number of cancer types [4], [17], [21]. In the case of the human endometrium, elevation of SRC expression levels has been correlated with endometrial hyperplasia and cancer [36]–[39] and with endometrial samples biopsied from patient groups predisposed to endometrial tumorigenesis [29]–[31]. Interestingly, our unpublished data show that knockdown of SRC-2 alone in Ishikawa human endometrial cancer cells significantly attenuates the ability of this cancer cell line to proliferate, supporting a role for SRC-2 in endometrial cancer cell progression. While our SRC-2:OE mouse has yet to exhibit endometrial cancer, the persistent hyperproliferative state observed in the epithelium of the SRC-2:OE endometrium may offer a favorable environment for the promotion of tumorigenesis initiated by an oncogenic event, a proposal which will be tested in the future.

In conclusion, we describe the generation of a new mouse model which allows for the perturbation of SRC-2 expression levels in a cell-specific manner. Apart from using this mouse as an investigative tool to disclose the molecular mechanisms that underpin the uterine phenotype resulting from SRC-2 overexpression, this mouse will be used to assess phenotypes in other target tissues in which the PR promoter is active, *i.e.* the mammary gland [46], [66]. Because the spatiotemporal specificity of targeted SRC-2 overexpression is controlled by the promoter driving cre expression, the SRC-2^LSL^ monogenic can be crossed with an appropriate cre mouse to examine the consequences of SRC-2 overexpression in other target tissues (*i.e.* prostate). Finally, we are currently using this mouse technology to generate similarly overexpressing mice for SRC-1 and SRC-3 in order to delineate the individual and integrative roles of these coregulators in pathological processes, such as occur in the endometrium.

## Supporting Information

Figure S1 Transcript levels of endogenous SRC/p160 family members are not significantly changed in the SRC-2:OE uterus. (A–C) Real time PCR analysis of transcript levels of mouse SRC-1, SRC-2, and SRC-3, respectively in SRC-2^LSL^ and SRC-2:OE uteri.
(TIF)

Figure S2 Immunohistochemical detection of myc-epitope tag in SRC-2:OE uterus. (A and B) Low magnification of myc epitope-tag immunostaining of transverse sections of the uterus from SRC-2^LSL^ and SRC-2:OE mice respectively. (C and D) represent high power magnification images of a sub-region shown in (A and B) respectively; S, LE, and GE denote stroma, luminal epithelium and glandular epithelium respectively. Scale bar in (A and C) apply to (B and D) respectively. (E) Gross morphology of the reproductive tract dissected from three month-old SRC-2^LSL^ and SRC-2:OE mice (representative of five mice per genotype). (F) Left histogram displays uterine/body weight ratios of SRC-2^LSL^ and SRC-2:OE mice; right histogram shows the average diameter length of the mid-section of the uterine horn from SRC-2^LSL^ and SRC-2:OE mouse groups (**denotes p<0.01 (n = 5 mice per genotype)).
(TIF)

Figure S3 A global knockout for SRC-2 in the mouse further demonstrates the specificity of the antibody used against human SRC-2 in immunohistochemical studies. (A–C) Transverse tissue sections of the mid-uterine horn immunostained for SRC-2 from the SRC-2^LSL^ control, SRC-2:OE (as shown in **Figure 4A and 4B**), and SRC-2 knockout (SRC-2KO) [11] mouse respectively. As expected, note the absence of SRC-2 immunopositivity in the SRC-2KO uterine section (panel C). Scale bar in (A) applies to all sections; LE, GE, S, and M denote luminal epithelium, glandular epithelium, stroma, and myometrium respectively.
(TIF)

Figure S4 Endometrial immunostaining for myc epitope-tag expression and BrdU incorporation. (A) Uterine sections from one SRC-2^LSL^ mouse and two SRC-2:OE siblings stained for myc epitope tag expression is shown (left panels represent low power magnification images; right panels are corresponding higher power magnification images (red box in left panels); LE and S indicate luminal epithelium and stroma respectively. Far right top panel shows a high power magnification image of the glandular epithelium (GE) from the SRC-2:OE endometrium stained for myc epitope-tag expression. The far right bottom panel shows high magnification image of the circular smooth muscle layer of the SRC-2:OE uterus stained for the myc-epitope tag; MC denotes myometrial cell (white and black arrowheads indicate cells negative and positive for myc epitope-tag expression respectively). Scale bar in top far right panel applies to bottom far right panel. (B) Serial sections of uteri shown in (A) stained for BrdU incorporation. Top panels show low power magnification images of representative transverse uterine sections from one SRC-2^LSL^ and one SRC-2:OE mouse stained for BrdU immunopositivity. Central (luminal epithelium) and bottom (glandular epithelium) panels represent corresponding higher power magnification images of sub-regions shown in the top panels. (C) Histogram displays percentage of luminal epithelial (LE) and glandular epithelial (GE) cells positive for BrdU incorporation (***denotes p<0.001 (n = 5 mice per genotype)).
(TIF)

Figure S5 Diverse signaling pathways altered in the decidualized uterine horn are unresponsive in SRC-2:OE mouse. To perform additional pathway analysis of decidual transcriptional changes, the cDNA was used as a substrate for Quantitative PCR Arrays (Qiagen, Valencia, CA). Expression changes in target genes of interest were confirmed by TaqMan. (A–C) Real time PCR analysis of genes representative of glucose metabolism, PI3K/Akt, and epithelial to mesenchymal transition are shown respectively.
(TIF)

Table S1 TaqMan Gene Expression Assays used in study.
(DOCX)
