# Correction: Perturbing the Cellular Levels of Steroid Receptor Coactivator-2 Impairs Murine Endometrial Function

**DOI:** 10.1371/journal.pone.0143652

**Published:** 2015-11-18

**Authors:** Maria M. Szwarc, Ramakrishna Kommagani, Jae-Wook Jeong, San-Pin Wu, Sophia Y. Tsai, Ming-Jer Tsai, Bert W. O’Malley, Francesco J. DeMayo, John P. Lydon

The following information is missing from the Funding section: This study was funded in part by a National Institutes of Health grant (HD-042311) to JPL.
