# Supplementary figures and images for: Perturbing the Cellular Levels of Steroid Receptor Coactivator-2 Impairs Murine Endometrial Function
Source: PLoS One. 2014 Jun 6;9(6):e98664. doi: 10.1371/journal.pone.0098664 (PMC4048228; doi:10.1371/journal.pone.0098664)

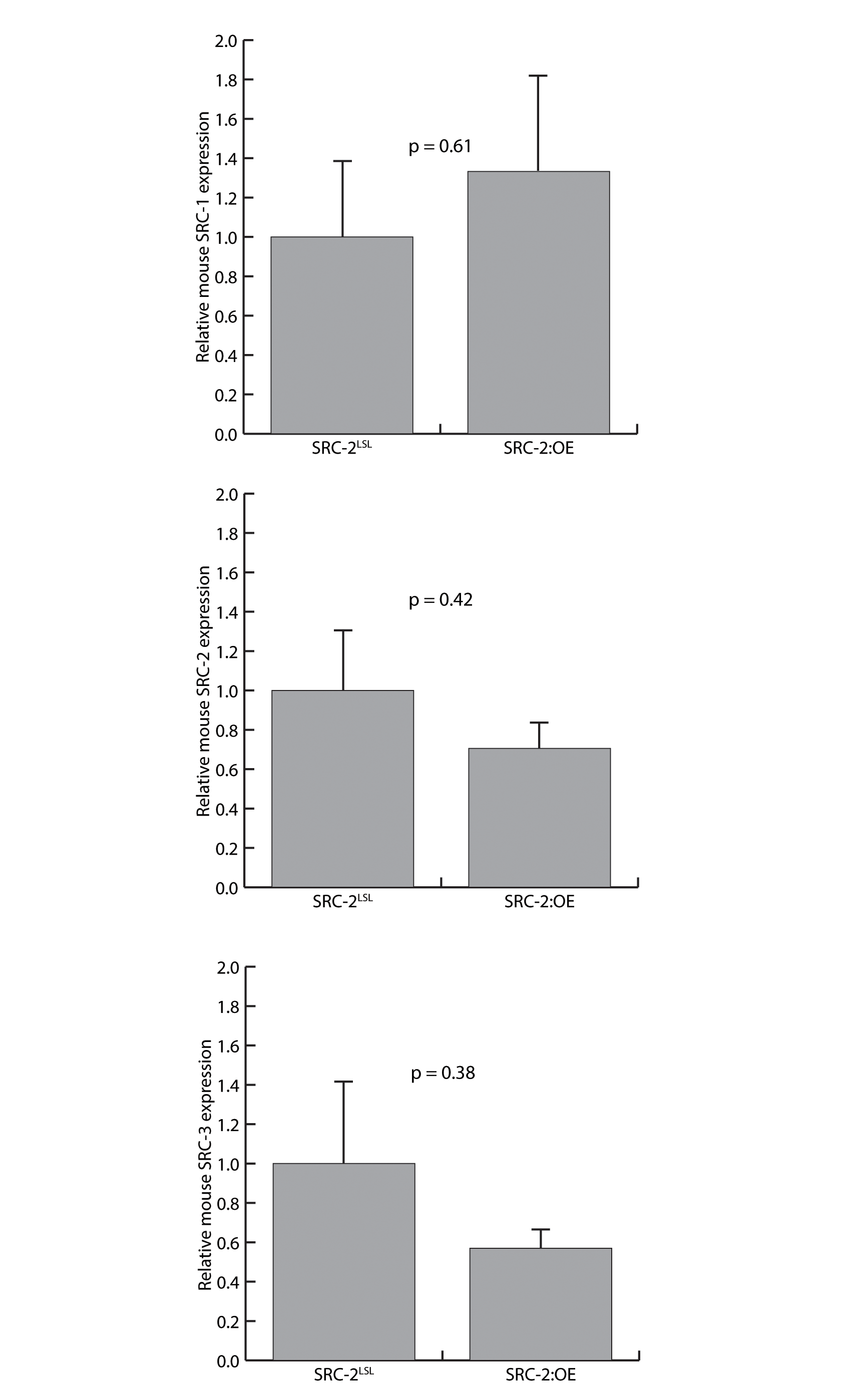

Supplement: Figure S1 — Transcript levels of endogenous SRC/p160 family members are not significantly changed in the SRC-2:OE uterus. (A–C) Real time PCR analysis of transcript levels of mouse SRC-1, SRC-2, and SRC-3, respectively in SRC-2LSL and SRC-2:OE uteri. (TIF) [file pone.0098664.s001.tif]

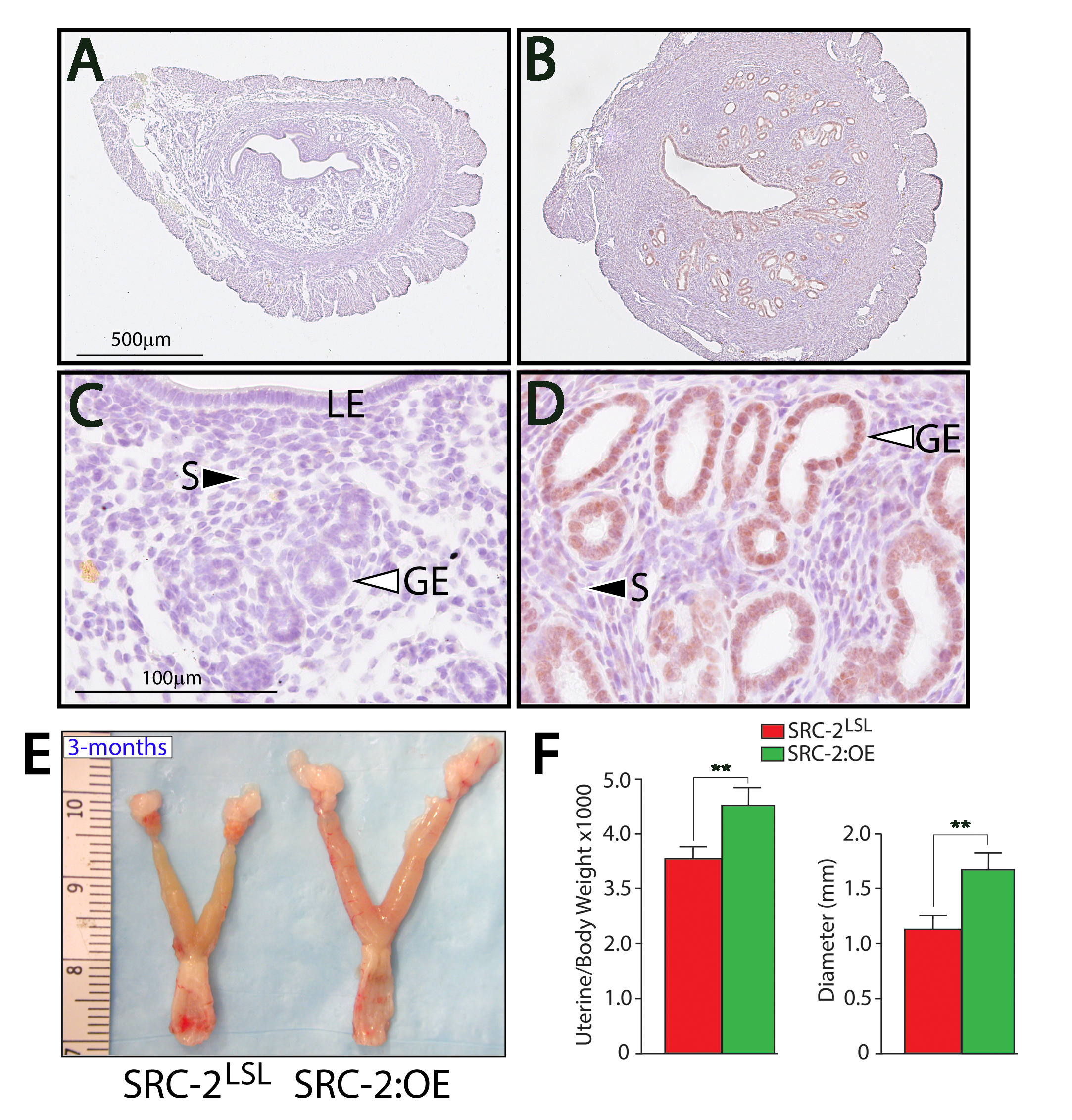

Supplement: Figure S2 — Immunohistochemical detection of myc-epitope tag in SRC-2:OE uterus. (A and B) Low magnification of myc epitope-tag immunostaining of transverse sections of the uterus from SRC-2LSL and SRC-2:OE mice respectively. (C and D) represent high power magnification images of a sub-region shown in (A and B) respectively; S, LE, and GE denote stroma, luminal epithelium and glandular epithelium respectively. Scale bar in (A and C) apply to (B and D) respectively. (E) Gross morphology of the reproductive tract dissected from three month-old SRC-2LSL and SRC-2:OE mice (representative of five mice per genotype). (F) Left histogram displays uterine/body weight ratios of SRC-2LSL and SRC-2:OE mice; right histogram shows the average diameter length of the mid-section of the uterine horn from SRC-2LSL and SRC-2:OE mouse groups (**denotes p<0.01 (n = 5 mice per genotype)). (TIF) [file pone.0098664.s002.tif]

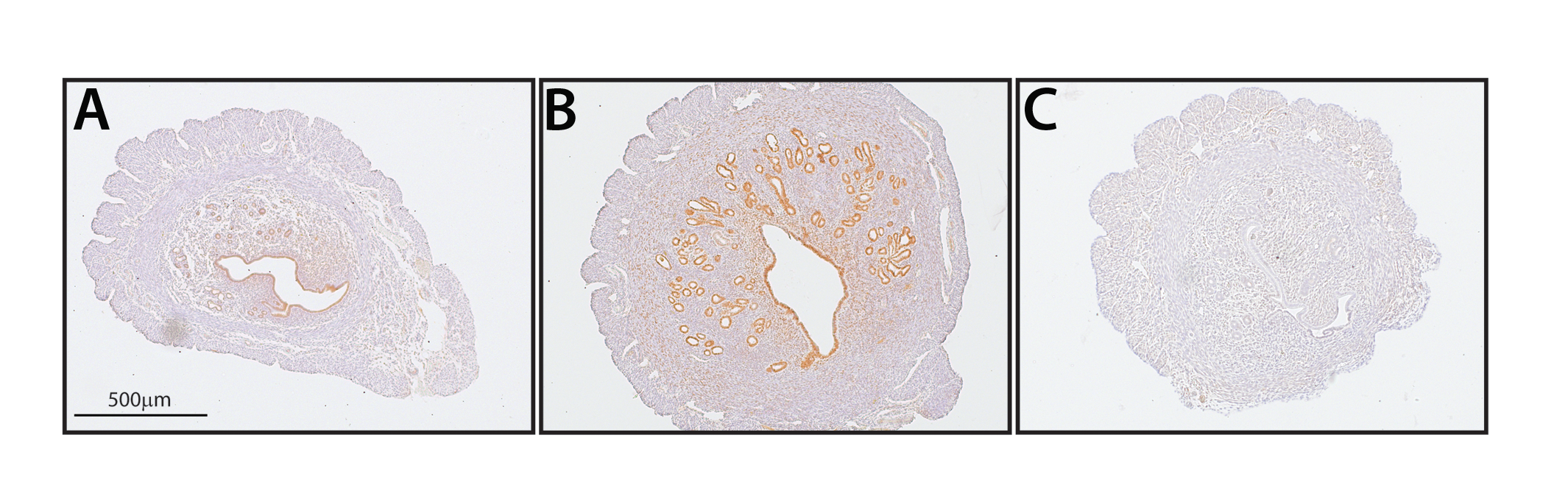

Supplement: Figure S3 — A global knockout for SRC-2 in the mouse further demonstrates the specificity of the antibody used against human SRC-2 in immunohistochemical studies. (A–C) Transverse tissue sections of the mid-uterine horn immunostained for SRC-2 from the SRC-2LSL control, SRC-2:OE (as shown in Figure 4A and 4B ), and SRC-2 knockout (SRC-2KO) [11] mouse respectively. As expected, note the absence of SRC-2 immunopositivity in the SRC-2KO uterine section (panel C). Scale bar in (A) applies to all sections; LE, GE, S, and M denote luminal epithelium, glandular epithelium, stroma, and myometrium respectively. (TIF) [file pone.0098664.s003.tif]

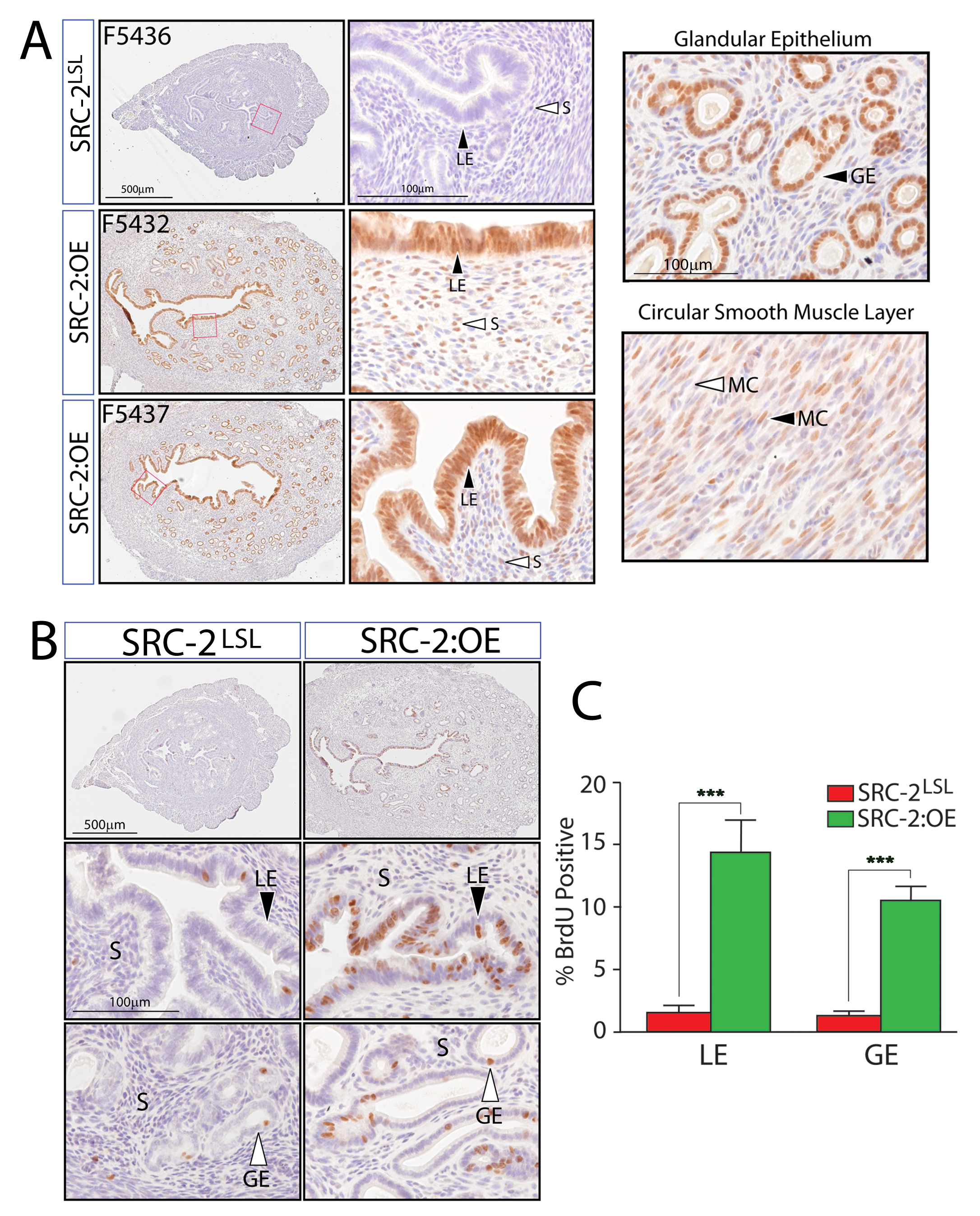

Supplement: Figure S4 — Endometrial immunostaining for myc epitope-tag expression and BrdU incorporation. (A) Uterine sections from one SRC-2LSL mouse and two SRC-2:OE siblings stained for myc epitope tag expression is shown (left panels represent low power magnification images; right panels are corresponding higher power magnification images (red box in left panels); LE and S indicate luminal epithelium and stroma respectively. Far right top panel shows a high power magnification image of the glandular epithelium (GE) from the SRC-2:OE endometrium stained for myc epitope-tag expression. The far right bottom panel shows high magnification image of the circular smooth muscle layer of the SRC-2:OE uterus stained for the myc-epitope tag; MC denotes myometrial cell (white and black arrowheads indicate cells negative and positive for myc epitope-tag expression respectively). Scale bar in top far right panel applies to bottom far right panel. (B) Serial sections of uteri shown in (A) stained for BrdU incorporation. Top panels show low power magnification images of representative transverse uterine sections from one SRC-2LSL and one SRC-2:OE mouse stained for BrdU immunopositivity. Central (luminal epithelium) and bottom (glandular epithelium) panels represent corresponding higher power magnification images of sub-regions shown in the top panels. (C) Histogram displays percentage of luminal epithelial (LE) and glandular epithelial (GE) cells positive for BrdU incorporation (***denotes p<0.001 (n = 5 mice per genotype)). (TIF) [file pone.0098664.s004.tif]

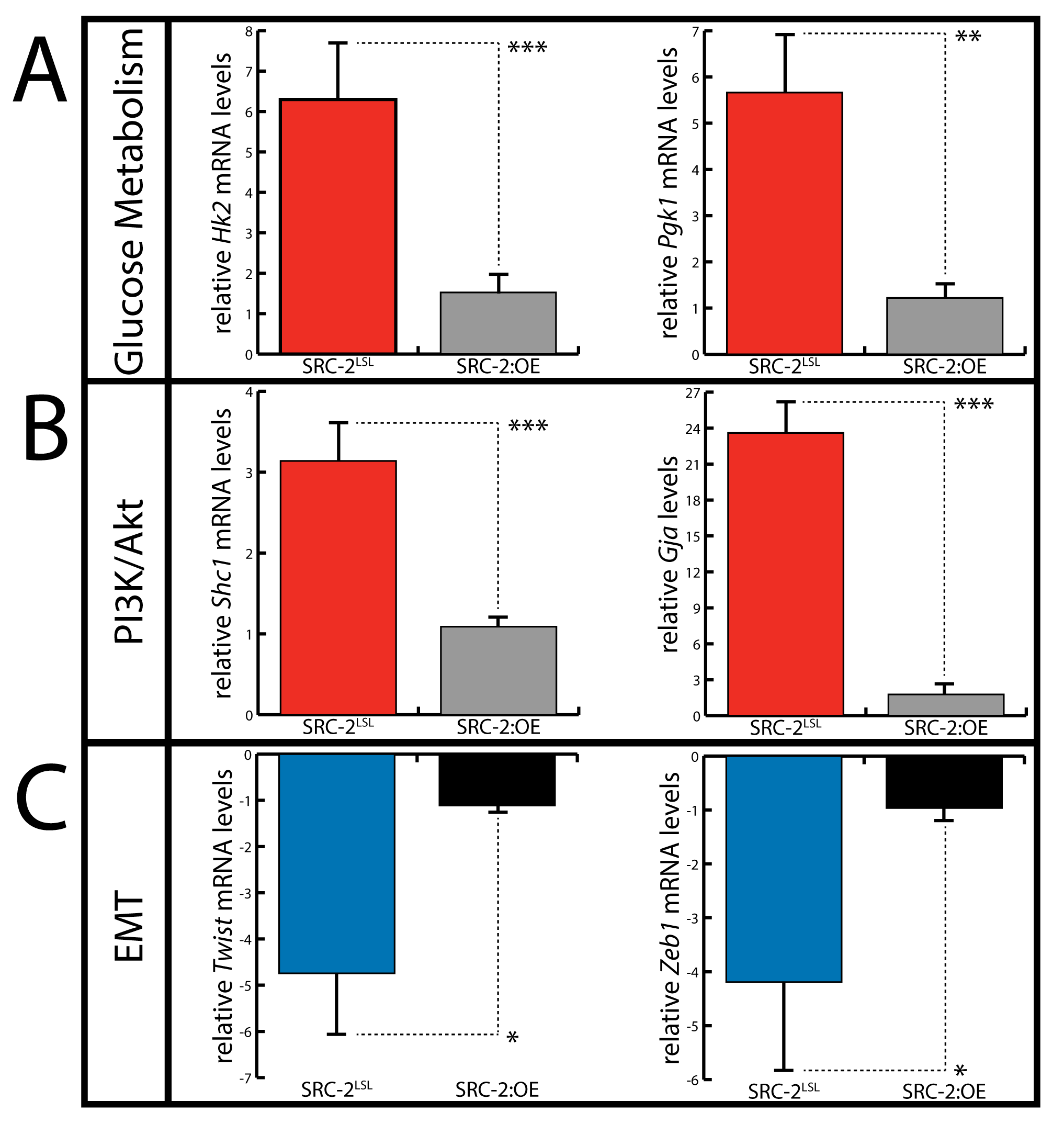

Supplement: Figure S5 — Diverse signaling pathways altered in the decidualized uterine horn are unresponsive in SRC-2:OE mouse. To perform additional pathway analysis of decidual transcriptional changes, the cDNA was used as a substrate for Quantitative PCR Arrays (Qiagen, Valencia, CA). Expression changes in target genes of interest were confirmed by TaqMan. (A–C) Real time PCR analysis of genes representative of glucose metabolism, PI3K/Akt, and epithelial to mesenchymal transition are shown respectively. (TIF) [file pone.0098664.s005.tif]
