# Supplementary material for: Perturbing the Cellular Levels of Steroid Receptor Coactivator-2 Impairs Murine Endometrial Function
Source: PLoS One. 2014 Jun 6;9(6):e98664. doi: 10.1371/journal.pone.0098664 (PMC4048228; doi:10.1371/journal.pone.0098664)
Supplement: Table S1 — TaqMan Gene Expression Assays used in study. (DOCX) [file pone.0098664.s006.docx]

Supplemental Table 1:

| **Gene** | **Assay Number** |
| --- | --- |
| Bmp2 | Mm01340178_m1 |
| Fst | Mm00514982_m1 |
| Gja1 | Mm01179639_s1 |
| Hand2 | Mm00439247_m1 |
| Hk2 | Mm00443385_m1 |
| Ncoa1 | Mm01318933_m1 |
| Ncoa2 | Mm00500749_m1 |
| Ncoa3 | Mm00500775_m1 |
| NCOA2 | Hs00896106_m1 |
| Pgk1 | Mm00435617_m1 |
| Shc1 | Mm00468942_g1 |
| Twist | Mm04208233_g1 |
| Wnt4 | Mm01194003_m1 |
| Zeb1 | Mm00495564_m1 |
